# Gut Microbiota: The Missing Link Between *Helicobacter pylori* Infection and Metabolic Disorders?

**DOI:** 10.3389/fendo.2021.639856

**Published:** 2021-06-17

**Authors:** Gracia M. Martin-Nuñez, Isabel Cornejo-Pareja, Mercedes Clemente-Postigo, Francisco J. Tinahones

**Affiliations:** ^1^ Unidad de Gestión Clínica de Endocrinología y Nutrición (Hospital Universitario Virgen de la Victoria), Instituto de Investigación Biomédica de Málaga (IBIMA), Universidad de Málaga, Málaga, Spain; ^2^ Centro de Investigación Biomédica en Red (CIBER) Fisiopatología de la Obesidad y Nutrición (CIBEROBN), Instituto de Salud Carlos III (ISCIII), Madrid, Spain; ^3^ Department of Cell Biology, Physiology and Immunology. Instituto Maimónides de Investigación Biomédica de Córdoba (IMIBIC)-Reina Sofia University Hospital, University of Cordoba, Córdoba, Spain

**Keywords:** *Helicobacter pylori*, gut microbiota, metabolism, eradication therapy for *Helicobacter pylori*, metabolic diseases, diabetes

## Abstract

*Helicobacter pylori* (*H. pylori*) is a gram-negative bacterium that infects approximately 4.4 billion individuals worldwide. Although the majority of infected individuals remain asymptomatic, this bacterium colonizes the gastric mucosa causing the development of various clinical conditions as peptic ulcers, chronic gastritis and gastric adenocarcinomas and mucosa-associated lymphoid tissue lymphomas, but complications are not limited to gastric ones. Extradigestive pathologies, including metabolic disturbances such as diabetes, obesity and nonalcoholic fatty liver disease, have also been associated with *H. pylori* infection. However, the underlying mechanisms connecting *H. pylori* with extragastric metabolic diseases needs to be clarified. Notably, the latest studies on the topic have confirmed that *H. pylori* infection modulates gut microbiota in humans. Damage in the gut bacterial community (dysbiosis) has been widely related to metabolic dysregulation by affecting adiposity, host energy balance, carbohydrate metabolism, and hormonal modulation, among others. Taking into account that Type 2 diabetic patients are more prone to be *H. pylori* positive, gut microbiota emerges as putative key factor responsible for this interaction. In this regard, the therapy of choice for *H. pylori* eradication, based on proton pump inhibitor combined with two or more antibiotics, also alters gut microbiota composition, but consequences on metabolic health of the patients has been scarcely explored. Recent studies from our group showed that, despite decreasing gut bacterial diversity, conventional *H. pylori* eradication therapy is related to positive changes in glucose and lipid profiles. The mechanistic insights explaining these effects should also be addressed in future research. This review will deal with the role of gut microbiota as the linking factor between *H. pylori* infection and metabolic diseases, and discussed the impact that gut bacterial modulation by *H. pylori* eradication treatment can also have in host’s metabolism. For this purpose, new evidence from the latest human studies published in more recent years will be analyzed.

## Introduction

*Helicobacter pylori* (*H. pylori*) is a microaerophilic spiral-shaped Gram-negative bacterium that can colonizes the stomach. This bacterium is able to generate an alkaline microenvironment that allows its survival in the acid gastric environment (1). The prevalence of *H. pylori* infection is more than 50% worldwide, but ranges from 19 to 88% depending on geographical area localization, with a higher prevalence in developing than in developed countries (2), which has been related to socioeconomic status and hygiene levels, including level of urbanization, sanitation, and access to clean water (2). However, other factors such as racial group could also be related to *H. pylori* prevalence (2, 3). The high prevalence of this infectious disease is a matter of concern due to the high number of *H. pylori*-associated clinical conditions.

*H. pylori* infection disturbs gastric homeostasis and, although most of the infected individuals remain asymptomatic, serious gastrointestinal complications are attributed to this bacterium such as peptic ulcer or chronic gastritis and promotes other life-threatening complications such as gastric cancer and mucosa-associated lymphoid tissue lymphomas (4). *H. pylori*-related diseases are not only restricted to the gastrointestinal tract, but *H. pylori* infection has also been associated with a number of extra-gastroduodenal disorders. In fact, there are solid evidence on its relationship with hematological disorders such as idiopathic thrombocytopenic purpura, iron deficiency anemia and vitamin B12 deficiency (5), but there is also a growing body of evidence that supports a link with neurological (e.g. Parkinson or Alzheimer disease, age-related cognitive decline), cardiovascular, liver, metabolic (e.g. diabetes, obesity, metabolic syndrome) and autoimmune and inflammatory (e.g. autoimmune gastritis, immune thrombocytopenia purpura, autoimmune thyroid diseases, inflammatory bowel diseases) disorders (6).

The precise mechanisms underlying the connection between gastric *H. pylori* infection and extra-gastroduodenal diseases remain unclear. The most accepted hypothesis to date is the generation of an inflammatory milieu due to the *H. pylori* insults to gastric mucosa and the consequent activation of innate and adaptive responses (1, 4). It has been hypothesized that this local inflammation at the stomach can spread systematically by the release of proinflammatory cytokines. This would favor the establishment of a low-grade and chronic inflammation, that is a common feature of *H. pylori*-associated extra-gastroduodenal disorders including cardiometabolic diseases such as diabetes, atherosclerosis or dyslipidemia (7, 8). However, this hypothesis has not been formally confirmed as yet, and knowledge about how inflammation actually links *H. pylori* with metabolic disorders is still insufficient.

Within this context, recent evidence showing that *H. pylori* infection not only disturbs the equilibrium of commensal bacterium species in the gastric mucosa, but it also leads to microbial changes in the human gut (9–18), has brought gut microbiota into the limelight (7, 9). Gut microbiota participates in host’s immune and metabolic homeostasis. In fact, dysbiosis of the gut bacterial community has been widely associated with obesity, diabetes and metabolic syndrome (19–21). Then, modulation of the gut microbiota during *H. pylori* infection could be triggering the onset and the impairment of metabolic disorders. Nevertheless, human studies addressing this issue are still scarce (10, 22–24).

Prophylactic recommendations to prevent *H. pylori* infection such as good household hygienic practices and the use of safe supplies of clean water are not always feasible, and while awaiting for an efficient *H. pylori* vaccine, the antibiotic therapy is the strategy of choice to fight against *H. pylori* infection at present (25, 26). Therapies for *H. pylori* eradication are based on the use of different antibiotic combinations (27–29) which are well known to have profound effects on the composition and diversity of the gut microbiota (30) and might also be related to adiposity and insulin resistance (31–34). Although an increasing number of studies have emerged analyzing the effects of the various antibiotic eradication therapies for *H. pylori* eradication on the composition of the gut microbiota (10, 11, 16, 17, 24, 35–44), few of them have related these changes with patients’ metabolic traits (10, 22–24). Within this context, our group has recently focused on analyzing how gut microbial changes promoted by *H. pylori* and its treatment can relate to metabolic traits.

In this review, we discuss the latest evidence from human studies on the influence of *H. pylori* infection and different eradication therapies on the composition of the gut microbiota, with particular focus on the relationship between *H. pylori*-induced gut microbiota modifications and host’s metabolic health.

## *H. pylori* Infection and Metabolic Diseases

Among the *H. pylori* infection-derived extra-gastric manifestations, the link with energy management, weight gain and metabolic homeostasis is still up for debate (6).

On the one hand, alterations in the gastrointestinal microenvironment due to *H. pylori* infection have been suggested to impair nutrient absorption and causes micronutrient deficiency. Malabsorption of iron and vitamins such as vitamin B12, vitamin A, vitamin C, vitamin E or folic acid have been related to *H. pylori* infection. Concordantly, associations between micronutrient deficiency complications and *H. pylori* infection have been found. For instance, the incidence of iron and B12 deficiency anemias is higher in *H. pylori* positive patients than in non-infected subjects (45, 46). Other disorders related to micronutrient deficiencies have been suggested to be associated with *H. pylori* infection, although further evidence would be necessary to confirm this hypothesis (45, 47, 48).

Moreover, lower levels of the orexigenic gastric hormone ghrelin (49), and higher levels of the anorexigenic adipokine leptin (50) have been reported in *H. pylori* infected patients. All together, these data led to propose that *H. pylori* infection can cause growth retardation and malnutrition in children. However, studies found that growth delay was also dependent on socioeconomic factors (8). Findings on weight management in adults during *H. pylori* infection are controversial with studies having reported positive (51–53), negative (54, 55) or non correlation (56, 57) between *H. pylori* infection and increased body mass index (BMI).

*H. pylori* eradication has been associated with weight gain likely due to the improvement of postprandial dyspeptic symptoms. In addition, changes in ghrelin and leptin levels after *H. pylori* treatment could be also acting on weight increase (58–60). An intervention study carried out in *H. pylori* positive children with previous growth retardation and low ghrelin levels, showed restoration of normal ghrelin levels and increased weight gain upon *H. pylori* eradication (61). By contrast, other studies reported significant weight loss, decrease in fat mass percentage and increase in fat-free mass after *H. pylori* eradication treatment (62, 63) or no effect on body weight (64) in adults. These contradictory results can be due to the different populations analyzed including different age groups. The influence of *H. pylori* treatment on body weight seems to differ in adults and children which could be due to age-related phenomena on body growth. In addition, other factors, such as gut microbiota, could be determining these discrepant results as discussed below.

On the other hand, imbalance of the aforementioned and other hormones such as glucagon-like peptide 1 (GLP-1), and the generation of a proinflammatory milieu might negatively affect metabolic homeostasis and weight in the adulthood (8). In fact, low-grade inflammation is a common feature of obesity, diabetes, insulin resistance, dyslipidemia and cardiovascular diseases (65).

Patients suffering of *H. pylori* infection are prone to display an unfavorable pro-atherogenic lipid profile featured by high triglycerides, total cholesterol and LDL-C and decreased HDL-C levels (22, 66–69). Zhao et al., found that, in addition to the dyslipidemic profile, bilirubin levels were also diminished in *H. pylori* infected patients (70). Bilirubin exhibits powerful antioxidant properties and has been inversely related to the risk of cardiovascular diseases and metabolic syndrome (71, 72). However, some studies did not find correlations between *H. pylori* infection and some of these lipid variables (73, 74). A recent meta-analysis of 27 previous studies concluded that most of the evidence tends towards an unfavorable lipid profile in *H. pylori* infected patients, although this is still casting doubt on the precise relationship with triglyceride levels (75). The causes of the altered lipid profile have not been elucidated, but impaired intestinal absorption and altered bile acid dynamics might be contributing to this condition (76). Notably, as discussed below, gut microbiota modulates these processes and could be acting as mediators in the establishment of the dyslipidemic profile in *H. pylori* patients. Supporting this hypothesis, antibiotic treatment for *H. pylori* eradication resulted in total and LDL cholesterol reduction and increase in HDL-C levels (77). Other studies described an elevation in total cholesterol levels at the expense of increasing HDL-C after *H. pylori* eradication (22, 78). However, the lipid improvement after *H. pylori* eradication was not confirmed by other authors (74, 78, 79). If considered the gut microbiome hypothesis, factors that can differentially modulate the gut microbial composition such as diet or the precise antibiotic combination used, might account for these discrepant results.

Associations between *H. pylori* infection and glucose homeostasis have been also explored (10, 23, 80, 81). Evidence suggests that *H. pylori* may be involved in both diabetes onset and impaired glycemic control in diabetic patients, but contradictory trends or lack of association between *H. pylori* infection and type 2 Diabetes have been also reported (80, 81). One of the mechanisms proposed for this relationship is the proinflammatory milieu induced by *H. pylori* infection that promotes gastric inflammation and cytokine secretion. In fact, *H. pylori* infection has been associated with increased levels of C-reactive protein (CRP) and interleukin 6 (IL6) (82, 83).

The link between *H. pylori* and Type 2 Diabetes was first analyzed in 1989 by Simon et al*.*, who found that the prevalence of *H. pylori* infection was higher in patients with Type 2 Diabetes compared to asymptomatic volunteers (84). Thenceforth, most of the studies have corroborated this finding reporting a higher prevalence of *H. pylori* in patients with Type 2 Diabetes (usually greater than 50%) in diabetic patients than in non-diabetic subjects (85–88). Recently published meta-analyses this year aimed at elucidating the direction of the relationship between *H. pylori* and Type 2 Diabetes (81). Pooled *H. pylori* prevalence in diabetic patients was 54%, but there were high (even contradictory) regional variability as the highest prevalence of *H. pylori* infection in patients with T2DM was 66% in Africa and the lowest was 15% in USA (81). Moreover, it was found that patients with *H. pylori* infection had a higher risk of Type 2 Diabetes, but results again differed depending on geographical regions with a direct relationship in Europe, Asian and Africa, but a negative relationship in USA (80). In subgroup analysis, the relationship between *H. pylori* and the risk of diabetes was different according to age, level of glycated hemoglobin A (HbA1c), duration of diabetes and methods for *H. pylori* detection. This suggests that these factors could be an important source of heterogeneity in the studies included in the meta-analyses (80, 81).

Furthermore, there are evidence suggesting that *H. pylori* infection is related to worse outcomes in diabetic patients. For instance, HbA1c levels were higher in diabetic patients with *H. pylori* infection in comparison with diabetic subjects negative for *H. pylori* (89). On the other hand, Yang et al. concluded that diabetic patients with *H. pylori* infection showed higher risk of cardiovascular diseases and more severe peripheral arterial stiffness than diabetic patients without *H. pylori* infection (90). Reciprocally, diabetic *H. pylori* positive patients showed worse symptomatology related to the infection (91, 92). A higher degree of insulin resistance was also reported in *H. pylori* infected patients from different populations (8), an association that was confirmed in every studies included in a systematic review but one (88).

Therefore, although the link between *H. pylori* infection and Type 2 Diabetes has been controversial in some studies, the general trend suggests a higher susceptibility to *H. pylori* infection in diabetic patients. Likewise, *H. pylori* infection would promote the development of Type 2 Diabetes, as well as worse glycemic control and insulin responsiveness, likely due in part to the enhanced inflammation. In this line, gut microbiota could be a contributing factor as a modulator of the inflammatory response as well as of the secretion of incretins involved in glucose homeostasis as detailed in next section. In this regards, some clues have been given from intervention studies based on the use of a cocktail of antibiotics for *H. pylori* eradication and found that non-diabetic patients with *H. pylori* infection subjected to antibiotic treatment, experienced an improvement in insulin resistance index, HbA1c and insulin levels concomitant to a reduction of low-grade inflammation (10, 87, 93). Of note, efficiency on eradication rates of infection was lower in diabetic patients than in non-diabetic (8). Higher rates of antibiotic resistance in diabetic patients than in non-diabetic ones might account for these differences (94). Furthermore, the improvement in glucose homeostasis, i.e. HbA1c and glucose levels, in diabetic patients after successful eradication of *H. pylori*, were not statistically significant in comparison with non-diabetic controls or baseline values (95). Notably, gut microbiota composition varies in diabetic patients as compared to non-diabetic subjects (96–98).

## Gut Microbiota, Antibiotic Therapy, and Metabolic Diseases

The human gut is colonized by a myriad of microorganisms that encompass bacteria, fungi, archaea, protozoa and viruses. This complex ecological community comprises symbiotic, commensal and pathogenic microbes that physiologically interact with the host at different levels and functions. Locally, gut microbiota contributes to the maintenance of gut barrier function and integrity, fermentation of indigestible dietary substrates, vitamin synthesis and immune system regulation. However, the activity of these microorganisms goes beyond the gastrointestinal tract, affecting the function of distant organs including brain, liver, pancreas, muscle or adipose tissue (99).

The impact that gut microbiota has on host’s metabolism is perhaps one of the most evident interrelations between the two systems. Besides harvesting energy from indigestible dietary fibers and modulating enterocyte function, gut bacterial community can also indirectly affect metabolic homeostasis by regulating immune system, local hormone secretion or bile acid synthesis. The efficiency of these actions depends on the specific and prevailing bacterial species that reside in the intestine (20, 100).

Several reports described differential bacterial profile in the gut from both obese and diabetic subjects (96–98). Although there are some controversial studies, in general terms, obesity-associated microbiota is depicted by an increase in *Actinobacteria* and *Firmicutes* and decline in *Bacteroidetes* phyla (97) as well as by diminished microbial richness and diversity (19, 32, 96, 101–103). Low microbial gene richness was associated with more adiposity, dyslipidemia and insulin resistance (101). In the same vein, diminished microbial gene richness has been related to low-grade inflammation and marked dysmetabolism (102) and, even among individuals with severe obesity, those patients with lower microbial gene richness had worse metabolic conditions (104). Among microbial genes associated with obesity, 75% belongs to *Actinobacteria* and 25% to *Firmicutes*, whereas 42% of lean-associated microbial genes belong to *Bacteroidetes* (96, 105, 106).

Gut microbiota composition from diabetic patients also differs from healthy subjects (107–109). Munukka et al., analyzed gut microbiota from women with and without metabolic disorders and concluded that the differences in some bacteria belonging to *Eubacterium rectal-Clostridium coccoides* group were more associated with obesity-related metabolic disorders than with obesity *per se* (110). Evidence also suggest that the amount of specific gut bacterial groups might also influence lipid metabolism. To be more precise, *Lachnospiraceae* was related to low LDL-C levels and both *Pasteurellaceae* and *Collinsella* associated with low triglyceride levels. *Tenericutes* and *Butyricimonas* were also related to a favorable lipid profile with low triglyceride and high HDL-C levels (111). Therefore, gut microbiota has been related to the different hallmarks of the metabolic syndrome.

These data point out the gut microbiota as a crucial player in the regulation of host’s energy homeostasis, though the mechanism by which the different bacterial species contribute to the regulation of metabolic functions are not fully understood. Current evidence point at low-grade inflammation and microbiota-derived metabolites as the possible links in the interplay of the gut microbiota and host’s metabolism. These topics have been extensively reviewed elsewhere (19–21, 112) and will only be briefly mentioned in this review.

As above mentioned, low-grade inflammation is a hallmark of obesity and its related metabolic complications. Inflammatory mediators interfere insulin signaling promoting insulin resistance. Immune cell infiltration in metabolic tissues impair its function and is enhanced in metabolic diseases. Concordantly, inflammation is related to diabetes, unhealthy lipid profile and favors the development of atherosclerotic lesions (113). However, the precise factors triggering the inflammatory response in metabolic diseases remains unclear. Within this context, it has been hypothesized that bacterial products, such as the Gram-negative bacteria cell wall component, lipopolysaccharide (LPS), can translocate from the gut to the circulation (114). LPS translocation would be enhanced by the consumption of fatty meals as these molecules are packed into chylomicrons in the enterocytes and delivered into the circulation (115). In addition, a “leaky” gut due to compromised gut barrier integrity would also favor LPS translocation (114). Circulating gut-derived LPS, that activate the inflammatory response by binding to its receptor TLR-4, can reach metabolic tissues such as adipose tissue and induce inflammation, insulin resistance and impaired lipid accumulation (116). The inability of adipose tissue to store excess energy results in increased blood lipids and toxic lipid accumulation into non-fatty organs such as liver or muscle (117, 118). In agreement with this hypothesis, circulating LPS levels are closely related to triglyceride levels and blood pressure, augment upon high-fat meal intake (115, 116, 119, 120) and its increase is associated with inflammation and lower expression of lipogenic markers in the adipose tissue of obese patients, as recently confirmed by our group (116).

During the fermentation of indigestible dietary substrates, intestinal bacteria produces different by-products such as short-chain fatty acids (SCFAs). SCFAs, such as butyrate, acetate and propionate, take part in a variety of physiological functions. SCFAs have been shown to regulate adiposity, to improve insulin sensitivity, to exert anti-inflammatory action by modulating immune cells and to regulate incretin secretion. In addition, these metabolites also participate in the maintenance of the gut barrier integrity, decreasing the risk of bacterial product translocation from the gut to the circulation (19, 21, 121–123). Butyrate represents a relevant energy source for colonocytes, contributes to epithelial cell health and consequently, to the integrity of intestinal epithelia. In addition, anti-inflammatory properties have been attributed to this SCFA (20, 124). Propionate stimulates the release of the satiety-inducing incretins GLP-1 and peptide YY, resulting in reduced food intake and concomitant weight, visceral adipose tissue and hepatic fat reduction, and preserved insulin sensitivity (125). Acetate is used by adipocytes, muscle or liver as energy substrate. It has been suggested that elevated levels of acetate and propionate associates with satiety, weight loss, and decreased inflammatory markers and blood lipids (126). However, some controversial results indicate that, when highly produced by gut microbiota, acetate could activates several pathways stimulating insulin secretion, hyperphagia and obesity in animal models (127). Then, the harmful or beneficial effects of SCFAs might be dependent on their levels as well as on the interaction with other factors (20). Notwithstanding this, human studies showed that obese and diabetic patients had diminished SCFA-producing bacteria and SCFAs (128). Therefore, SCFAs can mediate the multiple effect that gut microbiota exert on host´s metabolism.

Modulation of bile acid dynamics by gut microbiota has been also related to metabolic regulation (21). Bile acids participates in fat emulsification and absorption of lipids and liposoluble vitamins in the intestine. Primary bile acids are produced by hepatocytes, released in the proximal intestine, reabsorbed in distal ileum and recycled by the liver. Besides facilitating fat digestion and absorption, bile acids exerts metabolic functions by means of their receptors farnesoid X receptor (FXR) and Takeda G- protein receptor-5 (TGR5). FXR and TGR5 signaling promotes hepatic glycogen synthesis and insulin sensitivity, insulin secretion, energy expenditure in liver brown adipose tissue and muscle, thermogenesis and satiety (76). Gut microbiota metabolizes and deconjugates primary bile acids to transform them into secondary bile acids (129). Therefore, gut microbiota play a relevant role in determining the composition of bile acid pool which is relevant to the final biological actions of these molecules (130). In fact, it has been suggested that secondary bile acids can differentially interact with FXR and TGR5 than primary bile acid which would result in distinct metabolic actions (112, 131).

Obviously, bacterium by-products generated by the gut microbiota is highly determined by the diet. Bacteria are highly specific on the kind of nutrient they metabolize. Then, the effect of a certain type of diet on adiposity and metabolism activation not only is determined by calorie amount or energy harvesting by gut bacteria, but it is also defined by the specific kind of nutrient that enhances the growth of specific bacterial species damaging to coexisting microorganism by bacterial competition (99). For instance, some gut bacterial groups metabolized choline and L-Carnitine from dietary source (e.g. red meat or eggs) into trimethylamine (TMA). TMA is absorbed and oxidized by hepatic flavin monooxygenase 3 (FMO3) to produce trimethylamine *N*-oxide (TMAO) (132). Elevated levels of this metabolite have been described in diabetic (132) and obese (133) individuals and correlates with cardiovascular disease (134).

The production of these metabolites, the integrity of gut mucosa and consequently the actions that gut bacteria would exert as a whole, would depend on the abundance and proportion of each bacterial taxa inhabiting our intestine. Determinants of gut microbiota composition include dietary intake or antibiotic treatment, among other exogenous and endogenous factors (20, 30, 135, 136). Disruption of the gut bacterial equilibrium results in the deregulation of host metabolic functions and triggers the onset of obesity and obesity-related metabolic diseases (100, 112).

In this line, several studies aimed at assessing whether the modulation of the gut microbiota by antibiotic administration was related to obesity and other metabolic variables (31–34, 137, 138). Associations of diverse antibiotic treatments in early life with increased risk of childhood obesity has been reported (33, 34). A recent study focused on analyzing the gut microbiota profiles associated with the increased risk of childhood obesity due to early antibiotic exposure. This study showed that boys were at higher risk of increased abdominal adiposity than girls exposed to several courses of antibiotics and that changes in specific bacterial groups were related to both repeated antibiotic exposure and childhood adiposity (31). By contrast, Ajslev et al., described that, when mothers were overweight, early antibiotic exposure were related to decreased risk of developing childhood obesity. Unfortunately, gut microbiota was not analyzed in this study so it cannot be determined whether specific modifications in gut bacterial groups could account for the differential risk of developing obesity depending on maternal weight (137).

In the adult population, the association between antibiotic treatment, gut microbiota and insulin sensitivity is still controversial (32, 138). Vrieze et al., found that 1-week vancomycin treatment increased and decreased primary and secondary bile acids, respectively, and decreased insulin sensitivity evaluated 2-3 days after treatment cessation. However, amoxicillin administration had no effects on the study variables (32). Despite significant changes in gut microbiota diversity and composition as well as in SCFA and bile acids levels, Reijnders et al., did not find significant effects after 1-week vancomycin or amoxicillin exposure on different markers of insulin sensitivity, metabolic, hormonal or inflammatory parameters evaluated 8-weeks after treatment (138).

These findings point out the fact that factors such as age, gender, previous obesity degree, the type of antibiotic or post-therapy evaluation time could influence the effects on host’s metabolism. Therefore, further studies are required to elucidate how specific gut microbiota modulation exerted by each kind of antibiotic therapy affects metabolic homeostasis according to host’s characteristics. In view of the multiple therapeutic options for *H. pylori* eradication, this perspective become particularly relevant in the management of *H. pylori* infection. In addition, it is of interest to elucidate whether gut microbiota modulation by antibiotic eradication therapy improves or worsens the previous impaired metabolic status of *H. pylori* patients.

## Gut Microbiota in *H. pylori* Infection

*H. pylori* colonizes gastric mucosa leading to modification in the gastric microenvironment and disturbing gastric microbiota composition (139). These changes has serious local effects on stomach, but it also affects the function of other regions of the gastrointestinal tract which likely results in altered absorption of nutrients and drugs as well as on the production of incretins involved in metabolic homeostasis. Furthermore, few years ago, it was also proposed that *H. pylori* infection might also trigger large intestinal microbiota changes (140). Although close attention has been paid to the effect of *H. pylori* eradication treatment on gut microbiota, very recent human studies have also confirmed that *H. pylori* infection *per se* is linked to gut dysbiosis including alterations in bacterial diversity and abundance (9–18). A summary of bacterial shifts reported in individuals infected by *H. pylori* is displayed in **Table 1**.

**Table 1:** Human studies analyzing the impact of *H. pylori* infection on the gut microbiota determined by bacterial DNA sequencing.

Study (Reference)	Design	Methodology	Shifts in gut bacterial groups in HP+
Benavides-Ward et al. (15)	Paediatric Asymptomatic Peruvian population (Age=6-12 y). Study groups: HP+ (n=28) *vs* non-infected (n=28) children	Targeted sequencing	↑*Proteobacteria*, ↑*Firmicutes*, ↑*Clostridium*, ↑*Prevotella*
Chen et al. (17)	Adult Chinese population (age = 18-70 y). Study groups: HP+ (n=70) vs. *H. pylori*‐negative (n=35) subjects	16S rRNA V3-V4 region sequencing (MiSeq Platform)	↑*Sphingomonas* sp., ↑*Turicibacter* sp. ↓*Nitrospirae* ↓*Bacteroides plebeius*
Dash et al. (12)	Pooled Young and Adult Arabic population (Age=15.5-59 y). Study groups: Asymptomatic HP+ (n=12) *vs. H. pylori*‐negative (n=48) subjects	16S rRNA V4 region sequencing (MiSeq Platform)	↑Diversity ↑*Succinivibrio*, ↑*Turicibacter* ↑*Coriobacteriaceae*, ↑*Rikenellaceae* ↑*Desulfovibrio*, ↑*Enterococcaceae*
Frost et al. (13)	Adult Caucasian population (SHIP cohorts; Age= 43-63 y). Study groups: HP+ (n=212) *vs. H. pylori*‐negative (n=212) subjects	16S rRNA V1-V2 region sequencing (MiSeq Platform)	↑ Diversity ↑ *Haemophilus* ↓*Parasutterella*, ↓*Pseudoflavonifractor*
Gao et al. (14)	Adult Chinese population (Age=40-69 y). Study groups: HP+ (n=24) vs. *H. pylori*‐negative (n=15) subjects	16S rRNA V4 region sequencing (MiSeq Platform)	↑Diversity ↑*Bacteroidetes*, ↑*Firmicutes*, ↑*Proteobacteria*
He et al. (16)	Young Chinese population (Age=21-30 y). Study groups: Asymptomatic HP+ (n=17) *vs. H. pylori*‐negative (n=7) subjects	16S rRNA V3-V4 region sequencing (MiSeq Platform)	↑Diversity ↑*Proteobacteria*, ↑*Actinobacteria*, ↑*Acidobacteria*, ↑*Alistipes* ↓*Subdoligranulum*, ↓*Lachnoclostridium*
Iino et al. (18)	Adult Japanese population. Study groups: HP+ (n= 226) vs. *H. pylori*‐negative (n=524) subjects	16S rRNA V3-V4 region sequencing (MiSeq Platform)	↑*L. salivarius*, ↓*L. acidophilus*
Martin-Nunez et al. (10)	Adult Caucasian population (Age=18-65 y). Study groups: HP+ (n=40) *vs. H. pylori*‐negative (n=20) subjects.	16S rRNA V3-V4 region sequencing (MiSeq Platform)	↓Diversity ↑*Bacteroidetes*, ↓*Firmicutes* ↓*Actinobacteria* ↑*Bacteroidetes/Firmicutes* ratio
Wang et al. (141)	Adult Chinese population (Age=20-66 y). Study groups: HP+ (n=128) *vs. H. pylori*‐negative (n=185) subjects.	Shotgun metagenomic sequencing (BGISEQ-500 platform)	↑*Prevotella copri* ↑*Enterobacter cloacae* ↑*Klebsiella pneumoniae* ↓*Sutterella wadsworthensis* ↓*B. vulgatus* ↓*E. coli*
Wu et al. (11)	Adult Chinese population (Age=18-65 y). Study population: HP+ (n= 40) *vs. H. pylori*‐negative (n=20) subjects	16S rRNA V4 region sequencing (MiSeq Platform)	↓Diversity ↓*Actinobacteria*, ↓*Gemmatimonadetes*, ↓*Nitrospirae*, ↓*Chlorobi*, ↓*Thermi*, *WS3*, ↓*Caldithrix*.

The most significant bacterial groups showing differential proportions in H. pylori patients compared to non-infected patients are shown. HP+, H. pylori positive patients.

Within this context, our group and others have reported that human *H. pylori* infection is related to changes in bacterial diversity, but findings still controversial (10–14, 16). Some studies observed less diversity in *H. pylori* positive than in *H. pylori* negative subjects (10, 11). In view of the fact that *H. pylori* infection is usually related to unhealthy profile, these results are congruent with the general idea that high gut microbial diversity is an indicator of a healthy gut microbiome (101). In fact, low bacterial richness has been related to insulin resistance, dyslipidemia and higher overall adiposity (32). Nevertheless, other authors intriguingly found that several alpha diversity estimators exhibited higher scores, which indicate higher level of gut microbiota complexity in *H. pylori* cases (12–14, 16). Different study population, calculation of different diversity indexes or techniques for quantifying gut microbiota might underlie this opposite findings. In addition, other factors such diet or disease severity could be also influencing gut diversity. Therefore, controlled trials with homogenous designs are required to elucidate the actual effect of *H. pylori* infection and diversity of gut microbiota.

Regarding the specific groups of gut bacteria, some human studies have shown that the relative abundances of dominant phyla *Bacteroidetes*, *Firmicutes* and *Proteobacteria* significantly differ in the gut of *H. pylori* positive individuals compared to negative controls, although trends towards the increase, decrease or no differences have been reported (10, 14–16). Similarly, some studies found that *H. pylori* infection decreases the abundance of *Actinobacteria* (10, 11), but He et al*.*, reported an increase in this Phylum (16). Similar to bacterial diversity, variability in study population including age or geographical region and different exclusion and inclusion criteria might underlie the heterogeneity in major phyla proportion in *H. pylori* infected patients. In fact, it has been reported that gut microbiota can differ between Japanese and other populations (142). Differences in these phyla have been also related to obesity and Type 2 Diabetes (19). Microbial pattern with low *Bacteroidetes* and high *Actinobacteria* and *Firmicutes* proportions characterized the gut microbiota from obese patients (19, 96, 103). Therefore, further studies on *H. pylori* positive patients with more homogenous population might be helpful to establish consistent trends among predominant phyla in relation to metabolic diseases.

Despite not having found consistent results in the main phyla related to *H. pylori* infection, in general terms and taking into account the diversity of bacterial functions included in each phylum, bacterial changes at the level of the different taxonomic categories belonging to each phylum may have both positive and negative effects on the host. In this line, Frost et al., found that *H. pylori* infected individuals displayed elevated levels of the facultative pathogen *Haemophilus* but decreased levels of *Pseudoflavonifractor* and *Parasutterella* (13). *Pseudoflavonifractor* encompasses butyrate-producing bacteria and *Parasutterella* are succinate-producing bacteria (143). Thus, the decrease of *Pseudoflavonifractor* might partly explain the negative impact of *H. pylori* infection on metabolic profile as the result of reduced SCFA production. Notwithstanding this, the genus *Parasutterella* is increased in Crohn’s disease (144), so authors hypothesized that *H. pylori* might be exerting a protective role against pathogens related to other gastrointestinal diseases (13). In fact, succinate has been described as a virulence factor that might exacerbate enteric infections (145). In addition, this bacterium together with other taxa increased by *H. pylori* (e.g. *Alistipes*), have been proposed as potential predictor biomarkers of obesity-related metabolic abnormalities (146). In the same vein, succinate-producing bacteria and elevated levels of succinate have also been associated with Type 2 Diabetes and obesity (147).

By using shotgun metagenomic sequencing, Wang et al., found variation in other bacterial species according to the presence or absence of *H. pylori* infection (141). They specifically found increased proportions of *Prevotella copri*, a proinflammatory bacterium (148) as well as *Klebsiella pneumoniae* and *Enterobacter cloacae*, two infectious bacteria (149). These results suggest that *H. pylori* infection may promote growth of harmful bacteria in the gut. Conversely, *Sutterella wadsworthensis*, *B. vulgatus*, and *E. coli* amounts were lower in the *H. pylori*‐positive compared to non-infected individuals (141). Members of the genus *Sutterella* are highly prevalent commensals with the ability to adhere to intestinal epithelial cells indicating a possible immunomodulatory role (150). In addition, the pathobiont *Bacteroides vulgatus* has been implicated in the etiology of both Crohn’s disease and ulcerative colitis, but little is known about how its functional activity might drive the host inflammatory response (151).

A study carried out in a Japanese population specifically focused on analyzing how *H. pylori* modulates the proportion of each *Lactobacillus* species in the gut and found higher relative abundance of *Lactobacillus* in *H. pylori* infected patients than in non-infected individuals (18). *Lactobacilli* endows gastrointestinal tract with “colonization resistance” serving as a defense mechanism which protects the host from potentially pathogenic microbials. Probiotic effects have widely been attributed to different *Lactobacillus* species such as *L. casei* or *L. rhamnosus* that prevented antibiotic-related diarrhea (152), which may be due to the role of these bacteria in gut barrier preservation (153). Furthermore, certain *Lactobacillus* species have been also related to positive metabolic outcomes. While *Lactobacilli* relative abundance in the gut microbiota in obesity and after weight loss is controversial, it has been described that specific *Lactobacillus* strains (e.g. *L. rhamnosus*, *L. gasseri*, *L. plantarum* or *L. paracasei*) relates with a reduction of obesity-associated metabolic disorders and even an improvement on insulin sensitivity. However, other species such as *L. reuteri* has positively been associated with adiposity, which suggests *Lactobacillus* strain-dependent physiological effects on metabolism and weight regulation (19, 154–156). Iino et al., found that *H. pylori* positive patients displayed reduced amounts of *L. acidophilus* and increased proportion of *L. salivarius* in comparison with non-infected subjects. Authors suggested that alterations in *Lactobacillus* proportions could be related to the suppression of gastric acid secretion by *H. pylori* infection, but putative physiological effects that these changes can have on host’s metabolism and health have not yet been addressed (18).

In the same way that gastric colonization by *H. pylori* can affect the composition of gut microbiota, some gut bacteria might be also influencing the bacterial colonization of other gastrointestinal regions, including *H. pylori* in the stomach. In this regard, Chen et al., found that, despite not having noticing significant differences in the abundance of several putative beneficial taxa including *Bifidobacterium*, *Lactobacillus*, *Clostridium butyricum*, *Faecalibacterium prausnitzii* and *Akkermansia muciniphila*, the phylum *Nitrospirae* exclusively appeared in *H. pylori-*negative subjects (17). Similarly, in the study by Wu et al., this phylum presented low values in patients with duodenal ulcer and *H. pylori* infection (11). *Nitrospirae* are the most abundant and diverse nitrite-oxidizing bacteria which convert nitrite to nitrate (157). Of note, nitrite was shown to have bactericidal effects against *H. pylori* (158).

Differences between the gut microbiota from asymptomatic *H. pylori* infected patients *vs.* non-infected subjects have been also reported (12, 15, 16). In fact, *H. pylori* infection alters the gut microbiota in this asymptomatic patients by increasing *Proteobacteria*, *Clostridium*, *Firmicutes* and *Prevotella* in a paediatric population (15) and members belonging to *Succinivibrio*, *Coriobacteriaceae*, *Enterococcaceae*, and *Rikenellaceae* in adults (12) compared to non-infected subjects. The study by He et al., identified their study participants as asymptomatic although they also stated that subjects were diagnosed with superficial gastritis (16). To the best of our knowledge, it has not yet been evaluated whether the gut microbiota from asymptomatic *H. pylori*-infected patients also differs from that from symptomatic *H. pylori*-infected patients. This information could be of great interest to shed light on the possible role of the gut microbiota composition as player and/or biomarker of the severity of *H. pylori* infection which ranges from asymptomatic to serious clinical manifestation such as gastric cancers.

In this line of thought, Gao et al. investigated the association between the gut microbiota and *H. pylori*-related gastric lesions. To this end, they compared the intestinal microbiota of subjects without gastric injury, with gastritis or with intestinal metaplasia, and concluded that indeed the alterations of the fecal microbiota, particularly the phyla *Bacteroidetes*, *Firmicutes* and *Proteobacteria*, are likely involved in the process of progression of the gastric disease associated with *H. pylori* (14).

In addition, Frost et al., found that there were bacterial significant variations even within the *H. pylori* positive patient group that was dependent on the individual *H. pylori* antigen load. To be more precise, a negative association was reported between antigen load and *Bacteroides*, *Fusicatenibacter*, *Barnesiella*, and *Alistipes*, taxa exhibiting putative healthy characteristics at first sight. *Fusicatenibacter* and *Alistipes* are butyrate and lactate producing bacteria that could exert anti-inflammatory and other beneficial actions (159, 160). However, opposite results regarding different species belonging to *Alistipes* taxa and its relationship with dysfunction of several organs such as liver or cardiovascular system has been described (161). In fact, He et al., who also found that *H. pylori* infected patients had elevated levels of *Alistipes* compared to non-infected patients, referred to an animal study which found that this taxa was positively correlated with weight, fat mass, LPS and inflammatory cytokines (16, 162). On the other hand, *Barnesiella* is associated with more efficient eradication of antibiotic-resistant bacteria (163). By contrast, authors stated that non-significant results were retrieved from analyses taking into account *H. pylori* serology level (13). In the same vein, Iino et al., found that the relative abundance of *Lactobacillus* was significantly higher in *H. pylori* positive patients with severe atrophic gastritis as compared with infected patients with mild atrophy gastritis or without gastritis (18) supporting the hypothesis that gut microbiota could also differ depending on symptoms severity. Of note, no differences according to the degree of atrophic gastritis was found in the specific *Lactobacillus* species studied (*L. acidophilus* and *L. salivarius*) (18) which remarks the necessity to find the specific bacterial groups that can be related to infectious severity.

## Modulation of the Gut Microbiota by Antibiotic-Based *H. pylori* Eradication Therapies

According to the Kyoto consensus report, *H. pylori* gastritis has to be regarded as an infectious disease and the resulting recommendation is the treatment of all *H. pylori* infected patients regardless the clinical manifestations (164). Antibiotic therapies are the first-line treatment for *H. pylori* eradication, but the increasing antibiotic resistance rates make necessary the adaptation of the treatment regimen. In general terms, the standard triple therapy based on the combination of one proton pump inhibitor (PPI), clarithromycin and amoxicillin or metronidazole is recommended when *H. pylori* clarithromycin resistance is low (<15%). By contrast, quadruple therapy based on bismuth administration together two antibiotics and one PPI is the first-line treatment for *H. pylori* eradication in regions with high antibiotic resistance rates (27, 28). However, recent reports highlight how the increasing resistance to antibiotics compromises the efficacy of these recommended therapies and salvage regimens have to be used (28, 29). This lead to high variability on the type and amount of specific antibiotics that can have different impact on the patient’s health.

Antibiotic *H. pylori* therapies are not exempt from side effects. The extensively reported gastrointestinal adverse events associated to antibiotic administration such as nausea, vomiting or diarrhea among others, are linked to quantitative and qualitative changes in the gut microbiota (165). Antibiotic-induced changes in the gut microbiota has become a matter of concern in the treatment of *H. pylori* as the essential role of intestinal-residing bacteria in maintaining human health beyond gut function is increasingly accepted (27).

There is growing evidence that antibiotic-based therapies for *H. pylori* eradication promote alterations in the gut microbiota (10, 11, 16, 17, 24, 35–44), but different antibiotic combinations can exert differential effects on microbial community (166) (**Table 2**). In addition, proton pump inhibitors (PPI) which is usually administered together antibiotics for *H. pylori* eradication also contributes to bacterial shifts in the intestine (167).

**Table 2:** Effect of antibiotic *H. pylori* eradication therapies on gut microbiota composition in human studies.

Studies (Reference)	Design	Eradication therapy	Evaluation post-therapy	Changes in the gut microbiota after eradication therapy	Methodology
Jakobsson et al. (40)	Adult European population (Age =50-75 y). Comparison groups: HP+ *vs* post- eradication therapy (n=3).	PPI, amoxicillin, clarithromycin for 7 days.	8-13 days	↓ Alpha diversity, *↓Actinobacteria*, ↑*Enterococcus*, *↓Lachnospiraceae*, *↓Clostridia*, *↓Bifidobacteria*.	16S rRNA (454-based pyrosequencing) and TRFLP
			1 - 4 years	Alpha diversity restored, but microbiota composition NO returned to baseline.	
Oh et al. (41)	Adult Asian population (Average age = 49.3 y). Comparison groups: HP+ *vs* post- eradication therapy (n=10).	PPI, Clarithromycin, Amoxicillin for 14 days.	2 weeks	↓*Bacteroidetes*, ↓*Firmicutes*, ↓*Prevotella copri*, *↑Proteobacteria*.	16S rRNA (V1-V3). Roche/454 GS Junior platform
Yap et al. (42)	Young adult Asian population (Age= 18-30 y). Comparison groups: HP+ *vs* post- eradication therapy (n=17).	PPI, amoxicillin, clarithromycin for 7 days.	6 months	↓*Actinobacteria*, ↓*Proteobacteria* ↑*Verrucomicrobia*	16S rRNA (V3-V4). Miseq platform (Illumina)
			6 and 12 months	↑ *Firmicutes*, ↓*Bacteroidetes*	
			12 and 18 months	↑*Proteobacteria*, ↑*Actinobacteria*, ↑*Verrucomicrobia.*	
Yanagi et al. (24)	Adult Asian population (Age= 42-80 y). Comparison groups: HP+ *vs* post- eradication therapy (n=20).	PPI, amoxicillin, clarithromycin for 7 days.	1 week	↑*Bacteroidetes*, ↑Archaea, ↓*Actinobacteria*, ↓*Proteobacteria*.	16S rRNA. Miseq platform (Illumina),
			3 months	↑ B:F ratio, ↓*Actinobacteria*, ↓*Bifidobacterium*, ↑*Faecalibacterium prausnitzii*.	
Chen et al. (17)	Adult Asian population (Age= 18-70 y). Comparison groups: HP+ *vs* post- eradication therapy (n=35).	PPI, amoxicillin, furazolidone, colloidal bismuth pectin for 14 days.	14 days	↓*Firmicutes*,↓*Bacteroidetes*,↓*Verrucomicrobia* ↓*Lentisphaerae*, ↓B:F ratio, ↓*Lachnospiraceae*, ↓*Ruminococcaceae*, ↑*Proteobacteria*, ↑*Cyanobacteria* ↑*Klebsiella*.	16S rRNA (V3-V4). Miseq platform (Illumina)
			14 and 56 days	↑Enterobacteriaceae, ↑Leuconostocaceae, ↓Rikenellaceae, ↓Christensenellaceae, ↓Peptococcaceae, ↓Clostridiales Family XI, ↓Victivallaceae	
Gotoda et al. (43)	Adolescent Asian population (Age=14-15 y). Comparison groups: HP+ *vs* post- eradication therapy (n=8).	PPI, amoxicillin, clarithromycin for 7 days.	1 week	↓Alpha diversity, ↓*Actinobacteria*, ↓*Bifidobacteriales*.	16S rRNA (V3-V4). Miseq platform (Illumina)
			2 months	Microbiota returned to baseline.	
Hsu et al. (44)	Adult Asian population (Average age=48.8 y). Comparison groups: HP+ *vs* post- eradication therapy (n=11).	PPI, bismuth, metronidazole, tetracycline for 14 days.	2 weeks	↓Alpha Diversity, ↓*Bacteroidetes*,↓*Actinobacteria*, ↓*Verrucomicrobia, ↑Proteobacteria, ↑Klebsiella, ↑Morganella*, *↑Proteus, ↑Serratia*, *↑Escherichia*, *↑Cyanobacteria*, *↑Enterococcus, ↑Streptococcus*	16S rRNA (V3-V4). Miseq platform (Illumina)
			8 and 48 weeks	Microbiota returned to baseline. Alpha diversity restored at 48 weeks.	
Hsu et al. (35)	Adult Asian population (Average age=53 y). Comparison groups: HP+ *vs* post- eradication therapy (n=12)	PPI, amoxicillin, clarithromycin, metronidazole for 14 days.	2 weeks	↓ Alpha diversity*, ↓Bacteroidetes, ↓Firmicutes*, *↓Actinobacteria, ↑Proteobacteria*, *↑Escherichia, ↑Proteus, ↑Salmonella, ↑Klebsiella, ↑Morganella*.	16S rRNA (V3-V4). Miseq platform (Illumina)
			8 and 48 weeks	Microbiota composition and Alpha diversity returned to baseline.	
He et al. (16)	Asymptomatic Young Asian population (Age=21-30 y). Comparison groups: **a)** HP+ *vs* post- eradication therapy (n=10). **b)** Post- eradication therapy (n=10) *vs* controls (n=7).	PPI, bismuth, amoxicillin, furazolidone for 14 days.	6 weeks	**a)** ↓Alpha Diversity. No significant differences in phyla. **b)** No significant differences in Alpha diversity	16S rRNA (V3-V4) Miseq platform (Illumina)
			26 weeks	**a)** ↓Alpha Diversity, *↓Proteobacteria, ↓Bacteroidetes*, *↓Alistipes*, *↑Firmicutes, ↑Lachnoclostridium*, *↑Blautia*. **b)** Gut microbiota acquired a negative control-like profile.	
Liou et al. (36)	Adult Asian population (Age>20 y). Comparison groups: HP+ *vs* post- eradication therapy (n=408).	PPI, amoxicillin, clarithromycin for 14 days.	2 weeks	↓Alpha diversity, ↓*Fusobacteria*	16S rRNA (V3-V4). Miseq platform (Illumina)
			8 weeks and 1 year	Alpha diversity returned to baseline Microbiota returned to baseline.	
	Adult Asian population (Age>20 y). Comparison groups: HP+ *vs* post- eradication therapy (n=410)	PPI, amoxicillin, clarithromycin, Metronidazole for 10 days.	2 weeks	↓Alpha diversity, ↓Beta diversity, ↓*Bacteroidetes*, ↓*Firmicutes*, ↓*Fusobacteria*, *↑Proteobacteria*.	
			8 week and 1 year	↓Alpha diversity, Beta diversity restored at year 1. Microbiota returned to baseline.	
	Adult Asian population (Age>20 y). Comparison groups: HP+ *vs* post- eradication therapy (n=396).	PPI, bismuth, metronidazole, Tetracycline for 10 days.	2 weeks	↓Alpha diversity, ↓Beta diversity, ↓*Bacteroidetes*, ↓*Firmicutes*, ↓ *Fusobacteria*, *↑Proteobacteria*.	
			8 weeks and 1 year	↓Alpha diversity, ↓Beta diversity. Microbiota returned to baseline.	
Martin-Nunez et al. (10)	Adult European population (Age=18-65 y). Comparison groups: **a)** HP+ *vs* post- eradication therapy (n=40). **b)** Post- eradication therapy (n=40) *vs* controls (n=20).	PPI, clarithromycin, amoxicillin for 10 days.	2 months	**a**)↓ Richness, ↓*Actinobacteria*, ↓*Bifidobacteriaceae*, ↓*Bifidobacterium*, ↓*B*. *longum*, ↓*B*. *adolescentis*, ↓*Streptococcaceae*, ↓*Streptococcus* **b**) ↓Alpha diversity, ↑*Bacteroidetes*, ↓*Firmicutes*, ↓ *Actinobacteria*, ↓*Turicibacter*, ↓*Ruminococcaceae*, ↓Oscillospira, ↓*Oxalobacteriaceae*, ↓*Oxalobacter*, ↓*O*. *Formigenes*, ↓*Enterobacteriaceae*.	16S rRNA Miseq platform (Illumina)
Olekhnovich et al. (37)	Adult European population (Average age= 47.7 y). Comparison groups: HP+ *vs* post- eradication therapy (n=40).	PPI, bismuth, amoxicillin, clarithromycin + prebiotic for 14 days.	16 days	↓Alpha Diversity, ↓ *Actinobacteria*, ↓*Bifidobacterium adolescentis*, ↓*Verrucomicrobia*, ↑*Enterococcus faecium*, ↓Eubacteriaceae, ↓*Lachnospiraceae*, ↓*Ruminococcaceae*	Whole genome shotgun sequencing on ABI/SOLiD 5500W platform (Life Technologies)
Wu et al. (11)	Adult Asian population (Age= 18-65 y). Comparison groups: HP+ *vs* post- eradication therapy (n=20)	PPI, clarithromycin and amoxicillin for 14 days.	2 weeks	↓Alpha diversity, ↓*Tenericutes*	16S rRNA. Miseq platform (Illumina)
			4 weeks	↑*Bacteroidetes*	
			8 weeks	Microbiota returned to baseline.	
Kakiuchi et al. (39)	Adolescent Asian population (Age=15 y). Comparison groups: HP+ *vs* post- eradication therapy (n=31)	PPI, amoxicillin, clarithromycin for 7 days.	Therapy day 7 8-12 weeks after treatment	↓Alpha diversity, ↓*Collinsella*, ↓*Bifidobacterium.*	16S rRNA gene (V3-V4). Miseq platform (Illumina)
Tang et al. (38)	Adult Asian population (Age=18-65 y). Comparison groups: HP+ *vs* post- eradication therapy (n=74).	PPI, amoxicillin, furazolidone, bismuth potassium citrate for 14 days.	2 weeks	↓ Alpha diversity, *↑Proteobacteria*, ↓*Firmicutes*, ↓*Bacteroidetes*, ↓ *B:F, ↑Shigella, ↑Klebsiella, ↑Streptococcus, ↑Veillonella*, ↓*Bacteroides*, ↓*Faecalibacterium*, ↓*Roseburia*, ↓*Phascolarctobacterium*, ↓*Blautia*	16S rRNA gene (V3-V4). Miseq platform (Illumina)
			4 weeks	↓ Alpha diversity	
			6 and 8 weeks	Alpha diversity restored. No significant differences in phyla.	

The most significant and consistent changes in gut microbiota after the various H. pylori eradication therapies among human studies are summarized in this table. B:F, Bacteroidetes/Firmicutes ratio; PPI, proton pump inhibitor; TRFLP, terminal-restriction fragment length polymorphism.

In general terms, antibiotic administration has been related to a decrease in bacterial diversity (30, 138). Regarding the various *H. pylori* eradication therapies it has been reported a decrease in bacterial diversity after triple therapy consisting of PPI, amoxicillin and clarithromycin for 7 days (39, 40, 43), 10 days (10) or 14 days (11, 36). Nevertheless, most of these studies found that bacterial diversity was restored in the short and the long-term after treatment cessation (11, 36, 40, 43). However, other studies did not reported an improvement of bacterial diversity after cessation of triple therapy (10, 39).

Quadruple therapy with bismuth during 10 or 14 days also led to decreased bacterial diversity but not all the studies found a total recovery of bacterial diversity evaluated from 6 weeks to 1 year post-treatment (16, 36, 38, 44). By contrast, concomitant therapy of three antibiotics (amoxicillin, clarithromycin, metronidazole) without bismuth for 14 days decreased bacterial diversity, but it was fully (35) or partly restored (36) at 2 months or 1 year post-treatment, respectively.

Liou et al., compared the effect of triple, quadruple and concomitant therapies on bacterial diversity and found that the two latter treatments were related to lower alpha diversity in comparison with triple therapy at 1 year post-eradication treatment (36).

Notably, few studies have contrasted eradication therapy-induced bacterial diversity changes with bacterial diversity in the gut of non-infected patients (controls) which would drop hint at the similarity of bacterial diversity scores before and after eradication treatment to those seen in healthy conditions. However, opposite evidence has been found with no differences in the gut bacterial diversity at 6 and 26 weeks post-treatment with quadruple therapy compared to controls (16) or lower bacterial diversity evaluated at 2 months after triple therapy than in both controls and *H. pylori* infected patients before undergoing eradication intervention (10).

Pooled together, these findings suggest that the effect on gut bacterial diversity and its length are dependent on the eradication therapy used. A recent meta-analysis stated that alpha diversity was reduced immediately within the first week after eradication, but no consistent conclusions were drawn from studies evaluating bacterial diversity at longer evaluation times (168). It is worth of mention that this meta-analysis pooled different kinds of eradication therapies to analyze the effect on bacterial diversity, which could limiting the conclusions by adding variability to the results. This meta-analysis conclude that further studies will be required to gain more evidence before raising firm conclusions.

The effect of the diverse treatments used for *H. pylori* eradication has been also assessed in terms of bacterial abundance. Many studies have reported changes in specific bacterial taxa, but most of these findings remains controversial (10, 11, 16, 17, 24, 35–44). While some studies have observed a decrease in *Firmicutes*, *Bacteroidetes*, and an increase in *Proteobacteria* 2 weeks after triple (41), quadruple (17, 36, 38, 44) or concomitant (35) therapies, other studies have shown an increase in *Bacteroidetes* (11) and a decrease in *Proteobacteria* (11, 24) after triple therapy. The phylum *Actinobacteria* (24, 40, 43, 44) and members belonging to this phylum as *Bifidobacteriales* (43), *Bifidobacterium* (39), *Bifidobacteria* (40), and *Bifidobacterium adolescentis* (37) also remained decreased at 1 or 2 weeks post-treatment regardless of the therapy used. *Bifidibacterium* are regarded as beneficial bacteria for host’s health which promote gut barrier integrity, prevent gut mucosa colonization by opportunistic pathogens and are also involved in carbohydrate metabolism (169). SCFA producing bacteria such as *Lachnospiraceae*, *Ruminococcaceae*, *Eubacteriacea*, *Bacteroides*, *Faecalibacterium*, *Roseburia*, *Phascolarctobacterium* were also compromised at the short-term of the antibiotic treatment (**Table 2**). These bacteria might exert beneficial actions for the health as a consequence of the SCFA production. Concordantly, the decline of *Eubacteriaceae*, *Lachnospiraceae* and *Ruminococcaceae* has been associated with a broad spectrum of disorders (170).

On the contrary, the relative abundance of several putative detrimental bacteria which can release harmful factors for host’s health, such as *Escherichia*, *Proteus*, *Morganella* (35, 44), *Serratia* (44), *Klebsiella* (17, 35, 38, 44) and *Streptococcus* (38, 44) augmented upon antibiotic administration (quadruple or concomitant therapy) (**Table 2**).

Most of the studies tested whether antibiotic-induced changes in the abundance of bacterial groups were restored upon treatment cessation. As summarized in **Table 2**, gut microbiota composition is restored in most cases at 2 months post-treatment. Nevertheless, it has been also reported that the imbalance of some bacterial groups remains in the short- and the long-term after treatment cessation (10, 16, 24, 40, 42). Jakobsson et al., documented changes in the gut microbiota that persisted for up to 4 years after *H. pylori* eradication, but formal statistical analysis was not done owing to small sample size (40). In the same way, other studies also reported persistent changes in gut microbiota i.e. a decrease in *Proteobacteria*, *Bacterioidetes*, *Actinobacteria* and an increase in *Firmicutes* at 6 (16, 42) and 12 moths post-triple therapy (42), as well as an enrichment in *Proteobacteria*, *Actinobacteria*, *Verrucomicrobia* compared to baseline values at 18 months after treatment (42). Elevated relative *Bacteroidetes : Firmicutes* ratio was observed at 2 and 3 months of treatment cessation (10, 24). At phylum level, the meta-analysis made by Ye et al. showed a reduction in *Actinobacteria* and *Bacteroidetes* during the follow-up. In addition, *Firmicutes* was exclusively found to be augmented in the long-term after *H. pylori* eradication while *Poteobacteria* increased in short-term and returned to normality in the long-term (168). Lasting alterations in the proportions of these phyla may result in an altered production of bacterial metabolites which would affect host-bacterial crosstalk. For instance, acetate and propionate are mainly produced by *Bacteroidetes* members, while *Firmicutes* members typically produce butyrate (171). Notably, reduced *Bacteroidetes: Firmicutes* ratio and increased abundance of *Proteobacteria*, have been associated with obesity and the metabolic syndrome (19, 172).

Persistent antibiotic-induced shifts on bacterial groups can be associated with therapy adverse effects. In agreement with this, the relative abundance of the phylum *Proteobacteria* and some of its members including *Aggregatibacter* and *Sutterella* were higher at 2 weeks after quadruple therapy in patients who suffered from eradication intervention side effects compared with patients that did not reported adverse symptoms (44). *Proteobacteria* phylum includes many pathogens and it has been proposed that may be partly responsible for the development of adverse effects during eradication therapy (44).

Interestingly, some studies assessed the effect of probiotic supplementation administered together the antibiotic combination which attenuated the antibiotic-induced imbalance on gut microbiota composition (11, 41). Furthermore, probiotic supplementation have been associated with improved gastrointestinal symptoms (17, 39) and increased *Bacteroidetes:Firmicutes* ratio (17). All together, these effect might help to build up a beneficial gut microbiota profile after eradication therapy (38). This suggest that probiotic administration could attenuate antibiotic-induced gut dysbiosis, but to the best of our knowledge, it remains unexplored the consequences that this can have on host’s metabolic health within the context of *H. pylori* infection.

The discrepant observations between studies could be due to different eradication regimens, drug doses, treatment duration and/or sample size. Furthermore, other factors such as dietary habits, resistance to antibiotics and differences in the rate of absorption of antibiotics can affect the influence of eradication therapy on the intestinal microbiota and the time required to restore original bacterial composition. On the other hand, it is remarkable that most of the studies did not include controls without *H. pylori* infection, and considered that the gut microbiota was restored when no significant differences were observed regarding baseline. In this sense, it should be taken into account that pre-treatment bacterial composition is already influenced by *H. pylori* infection. This fact makes difficult to determine whether bacterial communities post-therapy mirror a healthy gut microbiota. All in all, further studies analyzing the effect of each eradication therapy and including non-infected controls, are necessary to clarify the effects that the various therapies for *H. pylori* eradication have on gut microbiota in the long-term and to elucidate the factors responsible for the variability in antibiotic response.

## Relationship Between Metabolic Traits and Gut Microbiota Modifications Induced by *H. pylori* Infection and Therapy

As detailed above, evidence has shown that *H. pylori* infection as well as its eradication treatment lead to gut dysbiosis. However, despite the fact that gut microbiota is closely linked to metabolic health, the role of gut microbiota in the relationship between *H. pylori* infection and metabolic dysregulation has been scarcely studied. Several studies from our group, which analyzed gut microbiota from *H. pylori* infected patients before and after eradication treatment (antibiotic triple therapy based on omeprazole, clarithromycin, amoxicillin) as well as non-infected control patients, addressed this issue (**Table 3**) (10, 22, 23). It was found that *H*. *pylori* eradication treatment produces specific bacterial shifts associated with changes in glucose homeostasis-related parameters [HbA1c, glucose area under the curve (AUC) calculated upon an oral glucose tolerance test (OGTT) (10)], GLP-1 (23), ghrelin (24) and HDL-C levels (22).

**Table 3:** Relationships between *H. pylori* eradication therapy-induced bacterial changes and metabolic variables.

Study (Reference)	Bacterial changes associated with metabolic variables post eradication	Putative bacterial functions	Variables
Martin-Nunez et al. (10)	(↓)*Rikenellaceae* (↓)*Megamonas* (↓)*Butyricimonas*	- Acetate, propionate and butyrate production. - Ability to degrade carbohydrates.	(↑)Glucose (AUC) (↑)HbA_1c_
Cornejo-Pareja et al. (23)	(↑)*Lachnobacterium* (↑)*B.adolescentis* (↑)*Coriobacteriaceae*	- Butyrate, acetate, and propionate production. - Ability to degrade carbohydrates. - Regulation of bile acid synthesis.	(↓)GLP-1 secretion
Martin-Nunez et al. (22)	(↓)*Delsufovibrio* (↓)*Rikenellaceae*	- LPS release - Acetate, propionate and butyrate production. - Regulation of CD36 expression	(↓)HDL levels
Yanagi et al. (24)	(↑) B:F	- SCFAs production - Bile acids - LPS release	(↓)Ghrelin levels

Summary of studies which have explored the association of the changes in gut microbiota composition after the administration of H. Pylori eradication treatment with metabolic variables. Arrows indicate the direction of the relationship between bacterial groups and clinical variables. AUC, Area Under the Curve; B:F, Bacteroidetes/Firmicutes ratio; GLP-1, glucagon-like peptide 1; HbA1c, glycosylated hemoglobin; HDL, high-density lipoprotein cholesterol; LPS, lipopolysaccharide; SCFA, Short-Chain Fatty Acid.

To be more specific, changes in the amount of *Rikenellaceae*, *Butyricimonas*, *E*. *biforme*, *B*. *fragilis*, and *Megamonas* were inversely associated with changes in glucose levels or glucose-related parameters, i.e. HbA1c, in *H*. *pylori* subjects after eradication treatment (10). Several studies have shown that these bacteria are involved in the fermentation of non-digestible carbohydrates and the generation of SCFAs such as acetate, propionate and butyrate (173–175). In addition, *Rikenellaceae* and *Butyricimonas* members are also able to use the environmental glucose to produce SCFAs, which could contribute to glucose level regulation (175, 176).

It was not only analyzed glucose metabolism, but also the dynamics of GLP-1 in the presence of *H. pylori* and upon antibiotic therapy (23). GLP-1, mainly produced in the ileum and colon by enteroendocrine L cells, regulates glucose homeostasis by systemic effects on pancreatic β cells, reduction of gastric acid secretion, delaying gastric emptying, regulating appetite as well as adipose tissue physiology (177, 178). Taking into account that some gut bacteria modulate intestinal enteroendocrine L cell secretion of GLP-1 (122, 179), it is of interest to explore whether both *H. pylori*-induced or antibiotic treatment-induced modifications in gut microbiota could also influence GLP-1 levels. In this way, GLP-1 levels at different time point after an OGTT before and after eradication treatment in *H. pylori* positive patients as well as in *H. pylori* negative controls were evaluated. We found that changes in GLP-1 AUC after eradication therapy positively and negatively correlated with the changes in the genus *Lachnobacterium and Bifidobacterium adolescentis*, respectively. Variation in GLP-1 at 60 min after OGTT and the changes in the family *Coriobacteriaceae* also positively correlated two months after the treatment (23). Species from the genus *Lachnobacterium* and *Bifidobacterium* are able to produce SCFAs (180). Among the beneficial effects attributed to SCFAs, these metabolites have also been proposed to favor GLP-1 L cell secretion and to exert anti-inflammatory action which may have beneficial effects on glucose homeostasis and insulin sensitivity (181, 182). Studies analyzing the use of *Bifidobacterium* as probiotic reported an increase in GLP-1 production and beneficial effects on carbohydrate metabolism (183). Concordantly, type 2 diabetic patients harbored smaller amounts of *B. adolescentis* (184). The cause and consequences of these opposite correlations between these two groups of gut bacteria and GLP-1 secretion in *H. pylori* positive patients after eradication treatment should be further analyzed. Bacteria belonging to the family *Coriobacteriaceae*, which has been shown to be reduced in Type 2 diabetic women (185), might also indirectly favors GLP-1 secretion by generating bile acids that stimulates GLP-1 secretion in L cells (19, 186).

Relationships between microbial groups and GLP-1 levels differed between *H. pylori* infected patients and non-infected controls. Positive correlations between the genus *Megamonas* [previously related to carbohydrate metabolism (187)] and GLP-1 levels were exclusively found in the control group. By contrast, bacteria belonging to the phylum *Proteobacteria* correlated positively with GLP-1 levels only in *H. pylori*-positive patients. Notably, these correlations disappeared after the eradication treatment and shifted to *Bifidobacterium longun* and the genus *Prevotella* which correlated positively and negatively with GLP-1 levels, respectively (23).

Other authors also explored the influence of *H. pylori* eradication therapy-related gut microbial changes on ghrelin levels (24). Ghrelin is a multifunctional hormone mainly secreted by gastric mucosa that regulates body weight by stimulating appetite, growth hormone secretion, fat storage among other relevant systemic functions in energy metabolism (188). Yanagi et al., reported that changes in the *Bacteroidetes:Firmicutes* ratio were inversely related to changes in plasma ghrelin levels after the administration of triple therapy for *H. pylori* eradication (24). Paradoxically, previous studies showed that modulation of the gut microbiota by using prebiotic supplementation reduced ghrelin secretion (123).

The relationship between specific profiles of gut bacteria in *H. pylori* infected patients before and after eradication treatment and metabolism are not restricted to carbohydrate metabolism but it seems to extend to lipid metabolism. Major phyla *Bacteroidetes* (increased in *H. pylori* positive patients) and *Firmicutes* (decreased in *H. pylori* positive patients) were negatively and positively correlated with HDL/LDL ratio, respectively. When analyzed in more detail, several bacterium taxa (*Eubacterium*, *Bacteroides coprophilus*, *E. biforme*), that were increased in *H. pylori* positive patients, were related to HDL/LDL ratio (22). Specific associations with lipid profile related to eradication treatment-induced gut microbial modifications were also found. Positive and negative changes in *Delsufovibrio* and *Rikenellaceae*, respectively, predicted changes in HDL-C levels at month 2 after completing antibiotic treatment (22). *Delsufovibrio* is a producer of LPS (189), but its products can also up-regulate the expression of the critical regulator of lipid absorption, CD36 (190), that has been positively associated with HDL-C levels (191). On the other hand, *Rikenellaceae* produces acetate that, besides the beneficial SCFA effects, has been described to promote hepatic *De novo* lipogenesis and cholesterol synthesis (192).

All in all, this emerging evidence suggest that gut microbiota shifts induced by *H. pylori* and upon antibiotic treatment for its eradication might, at least in part, underlie modulation of metabolic variables. However, further studies should be performed to confirm this hypothesis and to fill the many gaps of this intricate cross-regulation. Other *H. pylori* therapies than triple therapy should be assessed, as well as the concomitant effect of pre- or probiotic supplementation during eradication treatment. In addition, the precise mechanisms and bacterial activities involved in the *H. pylori*-gut microbiota-metabolism crosstalk remains to be elucidated.

## Discussion

While the number of studies analyzing the effect of *H. pylori* eradication therapies on the gut microbiota are increasing in recent years, current evidence is not enough to draw clear conclusions on the most relevant and common bacterial shifts. Future research needs to face several challenges to make clear assumptions on this relationship. Different population characteristics can lead to divergent results. Gut microbiota composition varies among ethnic groups, likely due to the different dietary, hygienic and genetic factors as well as regional antibiotic resistance rates (142). Thus, the modifications on specific bacterial taxa and the underlying related mechanisms involved in metabolic regulation could largely differs depending on the region where the study is performed. In this sense, the inclusion of non-infected healthy individuals as controls in intervention studies would give valuable information to determine the actual reversion degree to “healthy” baseline gut microbiome. Moreover, it is likely that the severity of the *H. pylori* eradication therapy impact on metabolism and adiposity in the long-term also varies depending on the age group. In that way, other antibiotic treatments than those used for *H. pylori* eradication have been shown to influence the onset of obesity when applied to early-life infants, while evidence on antibiotic administration effect on adult metabolism is not so clear (31–34, 137, 138). The fact that most of the studies analyzing the gut microbiota in *H. pylori* infected patients excluded subjects with diabetes, obesity and cardiovascular diseases (10, 16, 17) also limits the knowledge on the concomitant management of the dysbiosis induced by *H. pylori* infection and treatment in patients with these metabolic diseases.

Other unaddressed issue is how the different intestinal bacterial patterns influenced by infection severity relates to metabolic homeostasis. Furthermore, the relationship between *H. pylori*-induced changes in gut microbiome and metabolic disorders has been scarcely explored as yet, and the effect of each therapeutic options used to treat *H. pylori* infection as well as the concomitant use of probiotics on specific metagenomic and metabolic modifications should be also further addressed.

Finally, to the best of our knowledge, direct evidence on the bacterial functions and actions (by-product production, inflammation and translocation of bacterial products) resulting from the modulation of the diverse bacterial groups as a whole during *H. pylori* infection and treatment remains unexplored. This might be helpful to improving the understanding of the metabolic regulation by gut microbiota within the context of *H. pylori*-related disease.

In conclusion, although current data points at an essential role of gut microbiota as mediator of the crosstalk between *H. pylori* and host’s metabolic health, the numerous remaining unanswered questions warrant future in-depth research in this field.

## Author Contributions

MC-P and FJT contributed to conception and design of the manuscript. GMM-N, IC-P and MC-P researched the literature. GMM-N, IC-P, MC-P, and FJT drafted and wrote different sections of the manuscript and contributed to the discussion and interpretation of data. All authors contributed to the article and approved the submitted version.

## Funding

GMM-N was supported by a Juan de la Cierva, Formación contract (FJCI-2017-34349; Ministerio de Ciencia, Innovación y Universidades; Spain). MC-P was recipient of a postdoctoral grant Juan de la Cierva Formación (FJCI-2017-32194) from the Ministerio de Ciencia, Innovación y Universidades (Spain) and a postdoctoral research grant (DOC_00448) from the Consejeria de Economía, Industria, Conocimiento y Universidades (PAIDI, Junta de Andalucía, Spain), co-funded by the Fondo Europeo de Desarrollo Regional (FEDER). IC-P has been recipient of postdoctoral grants Río Hortega (CM 17/00169) and Juan Rodés (JR19/00054) from the Instituto de Salud Carlos III (ISCIII) and co-funded by Fondo Europeo de Desarrollo Regional (FEDER). This research was funded by Centros de Investigación Biomédica en Red (CIBER, CB06/03/0018) from the ISCIII, (Madrid, Spain); RIC-0539-2018 and PI-0092-2017 from Consejería de Salud (Junta de Andalucía, Spain); PI18/01160 from the ISCIII (Madrid, Spain), and co-funded by the Fondo Europeo de Desarrollo Regional (FEDER).

## Conflict of Interest

The authors declare that the research was conducted in the absence of any commercial or financial relationships that could be construed as a potential conflict of interest.
